# Psychometric Properties of the World Health Organization Quality of Life Scale for Older Adults (WHO-QoL-Old) in a Mexican Population

**DOI:** 10.3390/geriatrics9050134

**Published:** 2024-10-14

**Authors:** Christian Díaz de León Castañeda, Ana Celia Anguiano-Morán, Elva Rosa Valtierra-Oba, Barbara Monica Lemus-Loeza, Gabriela Galván-Villalobos, Alaín Raimundo Rodríguez-Orozco

**Affiliations:** 1 Consejo Nacional de Humanidades, Ciencias y Tecnologías (CONAHCYT), Ciudad de México 03940, Mexico; christian.diaz.de.leon@umich.mx; 2 Facultad de Enfermería, Universidad Michoacana de San Nicolás de Hidalgo, Morelia 58260, Mexico; ana.anguiano@umich.mx (A.C.A.-M.); elva.valtierra@umich.mx (E.R.V.-O.); barbara.lemus@umich.mx (B.M.L.-L.); 3 Facultad de Ciencias Médicas y Biológicas “Dr. Ignacio Chávez”, Universidad Michoacana de San Nicolás de Hidalgo, Morelia 58020, Mexico; gabriela.galvan@umich.mx

**Keywords:** quality of life, older adults, psychometrics, confirmatory factor analysis

## Abstract

Background/Objective: The present study aimed to contribute to analyzing the psychometric properties of the WHO Quality of Life Scale for Older Adults (WHO-QoL-Old) in a sample of older adults in Michoacán, Mexico. Methods: 111 older adults from Michoacán, Mexico, participated in the study. Confirmatory factor analysis (CFA) was conducted to test the fit of various models. Data analysis was performed using R Studio, considering the ordinal nature of the items in the model estimation method. Internal consistency was evaluated using the alpha coefficient (α) and McDonald’s omega coefficient (ω). Results: The CFA indicated that the six-correlated-factor model proposed theoretically showed a very good fit (χ^2^: 397.11, *p* < 0.001; CFI: 0.958; SRMR: 0.079; RMSEA: 0.079). The factors within the model demonstrated acceptable internal consistency, with an alpha coefficient ranging from 0.739 to 0.874 and an omega coefficient ranging from 0.748 to 0.882. Conclusions: It is concluded that the WHO-QoL-Old scale presents good psychometric properties for the Mexican older adult population.

## 1. Introduction

In the context of the demographic and epidemiological transition experienced by the Latin American and Caribbean region and many countries worldwide, monitoring older adults’ care and living conditions is of significant importance. Global disease burden statistics reveal the vulnerability of this age group through indicators such as Years Lived with Disability (YLD), a metric established by the Institute for Health Metrics and Evaluation (IHME). According to this indicator, in 2019 (before the COVID-19 pandemic), the Latin American and Caribbean region recorded 27,726 YLDs per 100,000 inhabitants for adults of both sexes aged 70 and older. The ten leading causes of disability and their respective percentage contributions were (1) hearing loss (10.2%); (2) type 2 diabetes (9.4%); (3) lower back pain (7.4%); (4) Alzheimer’s disease and other dementias (6.1%); (5) edentulism and tooth loss (4.7%); (6) other musculoskeletal disorders (4.6%); (7) falls (4.0%); (8) major depression (2.8%); (9) knee osteoarthritis (2.7%); and (10) cataracts (2.7%) [1].

The statistics mentioned above show the high burden of disabling diseases that compromise the quality of life of older adults. In light of this problem, it is essential to employ instruments that assess the quality of life of older adults in its various facets, have adequate psychometric properties, and are culturally adapted. Quality of life has been defined in various ways by different sciences and disciplines, as it involves multiple dimensions, namely that it is a multidimensional construct [2]. The World Health Organization (WHO) has defined quality of life as “the perceptions of individuals regarding their position in life in the context of the culture and value systems in which they live and in relation to their goals, expectations, standards, and concerns” [3].

Various instruments have been developed for measuring quality of life, some of which are generic, meaning they can be applied across different age groups and health conditions, while others are specific to particular age groups and health conditions [4]. Furthermore, instruments for evaluating quality of life can be divided into two groups: (1) those focused on assessing health-related quality of life (HR-QoL), which primarily evaluate physical and mental functionality and most of which incorporate the assessment of individual preferences, thereby enabling the calculation of utility (a metric important for economic evaluations); and (2) those focused on assessing well-being, a broader construct that includes not only physical and mental dimensions but also aspects such as autonomy, social participation, relationships, and personal projects, etc. Most of these latter instruments do not incorporate the assessment of preferences, thus precluding the calculation of utility [5].

The conceptualization and integration of the quality of life construct may vary for different age groups and health conditions. For instance, several important dimensions or facets have been identified specifically for older adults [6]. Moreover, specific quality of life assessment instruments have been developed for older adults, including preference-based instruments, such as the ICEpop CAPability Measure for Older People (ICECAP-O), and non-preference-based instruments, such as the following: the Older People’s Quality of Life (OPQOL) instrument; the Control, Autonomy, Self-realization, and Pleasure (CASP 19) instrument; and the WHO Quality of Life Instrument-Older Adults Module (WHO-QoL-Old) [7].

The WHO-QoL-Old instrument is specifically designed to assess the quality of life from a well-being perspective and is a specialized submodule that addresses the need for scales tailored to older adults [8,9]. This submodule was intended to be used complementarily with broader and more generic quality of life scales, namely the WHO-QoL-100 and WHO-QoL-Bref, which also adopt a well-being approach [9].

Like its predecessors, the WHO-QoL-Old scale was originally developed in English and consisted of a total of 24 items distributed across six factors: (1) Sensory abilities; (2) autonomy; (3) past, present, and future activities; (4) social participation; (5) death and dying; and (6) intimacy. Each factor has four items, and each item has five response categories, although the nomenclature for these categories may differ depending on the item and factor. Additionally, seven items are presented in reverse order, meaning it is necessary to reverse them when obtaining the scores for the factors and the total scale [8,9]. It should be noted that proposals have also been made for one-dimensional shorter versions of the scale [10].

Since its development, the WHO-QoL-Old scale has performed very well, with evidence supporting its validity and reliability across various countries [8,9]. Additionally, further studies have been published that examine the validity of the scale in different languages, incorporating new analytical tools such as confirmatory factor analysis (CFA) and even item response theory (IRT) models [11,12,13].

The Spanish version of the WHO-QoL-Old scale was initially applied in Uruguay as part of its original development, yielding favorable results [8]. Subsequently, a study conducted in Spain performed a construct validity analysis based on the correlations between the factors of the same scale and the factors of the WHO-QoL-Bref, as well as validity based on relationships with other variables (convergent validity and discriminant validity) [14]. Another study conducted in Peru used CFA to demonstrate a good fit for both the original model, which was comprised of six correlated factors, and one of the previously proposed unidimensional short versions [15]. 

Several adaptations of the WHO-QoL-Old scale have been applied in Mexico to contribute to the study of its psychometric properties. One study conducted in Mexico analyzed the validity based on the internal structure of the scale via exploratory factor analysis (EFA) based on principal component analysis (PCA). It also evaluated its internal consistency (alpha coefficient) and the relationship with other variables, such as depression and quality of life, as assessed using other psychometric instruments, yielding satisfactory results [16]. Although another study also conducted this factorial analysis, it failed to confirm the factorial structure originally proposed by the WHO-QoL-Old scale [17].

A notable adaptation of the WHO-QoL-Old scale in Mexico modified some items to fit the Mexican context better and included images or pictograms to illustrate the response categories for the items. This adaptation also involved performing a PCA-based EFA, analyzing an approach of reliability, and the relationship with other variables, yielding satisfactory results [18]. A subsequent study conducted in Peru used this adapted scale and found evidence supporting its validity based on its internal structure, reliability, and relationship with other variables [19].

Another recent study, also conducted in Peru, explored the internal structure of the WHO-QoL-Old scale using CFA to test both the six-factor correlation model and a bifactor model. However, the model estimation methods did not consider the ordinal nature of the items, while the theory originally proposed by the scale was not verified, and several items had to be removed to obtain good results [20].

Returning to the Mexican adaptation of the WHO-QoL-Old scale, in which visual diagrams are added to support participants’ response to the items, it is noteworthy that this modification can be of great help for older adults, in particular those with visual impairment, thus underlining the importance of such modalities for instruments targeting this age group. As studies conducted to date on this adaptation have explored validity based on the internal structure using EFA and PCA, it presents an opportunity for research in this field via CFA and estimation methods that consider the ordinal nature of the items, the use of which has been suggested in the psychometric research literature [21].

The present study aims to contribute to studying the psychometric properties of the Spanish version of the WHO-QoL-Old scale, as adapted in Mexico for older adults.

## 2. Materials and Methods

### 2.1. Participants

A total of 111 older adults participated in the present study, which was conducted in 2023. Participants were either residents in care institutions (nursing homes) or members of diurnal care centers for older adults in Morelia, Michoacán, Mexico, during the study period. Table 1 presents the main socio-demographic characteristics of the participants.

### 2.2. Instruments

The present study used the Spanish version of the WHO-QoL-Old scale, adapted in Mexico to incorporate iconography that graphically represents the categories of response to the items [18,19].

To analyze the relationship with other variables, the present study used the Spanish versions of the Geriatric Depression Scale (GDS), consisting of 30 dichotomous items with yes or no responses [22], and the Hamilton Anxiety Scale (HAS), consisting of 14 items with five response levels [23].

For the above research aim, a structured questionnaire was designed to include items exploring the socio-demographic variables of the participants and the 24 items of the Mexican adaptation of the WHO-QoL-Old scale (including the pictograms for the response options) and both the GDS and HAS scales. The questionnaire was delivered to the study participants in print form to be filled out by them, with support provided for this if necessary.

### 2.3. Data Analysis

#### 2.3.1. Confirmatory Factor Analysis (CFA)

**Figure 1:**
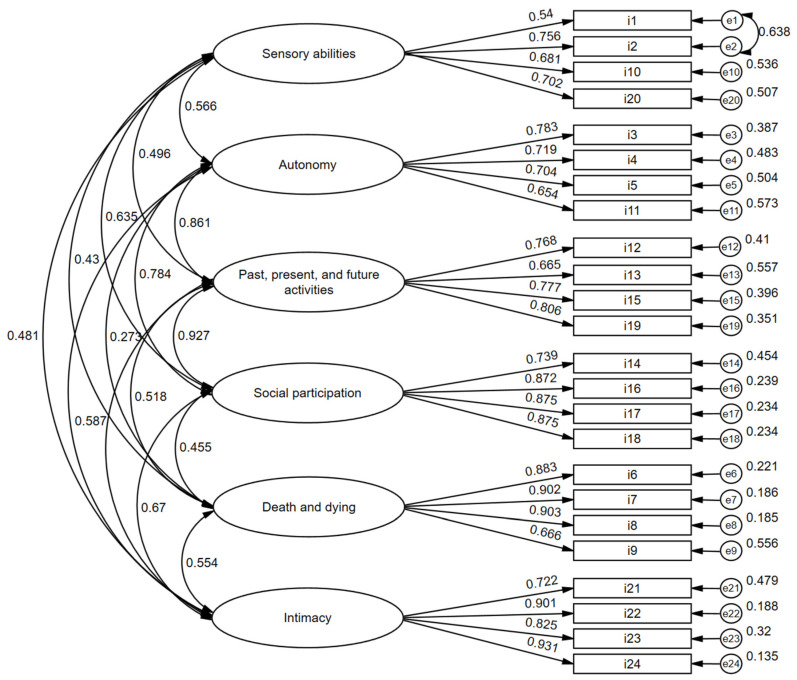
Results of the CFA conducted on the six-correlated-factor model for the WHO-QoL-Old scale.

A CFA was conducted to test different models of the WHO-QoL-Old scale, including a one-dimensional model and the six-factor correlated model (Figure 1). The application of the CFA used the estimation method WLSMV (weighted least squares mean and variance), which considers the ordinal nature of the items [21]. Adjustment indices were obtained as the following: statistical chi-square (2); comparative fit index (CFI, acceptable adjustment criterion > 0.90); standardized root mean square residual (SRMR, acceptable adjustment criteria < 0.080); and root mean square error of approximation (RMSEA, acceptable adjustable criteria < 0.80). The effect of parameter release, namely the covariances between the errors of the items as identified by the best index of modification, was tested in the models evaluated. 

#### 2.3.2. Reliability

As a reliability analysis approach, internal consistency was evaluated by calculating the α and ω coefficients for the six factors of the model proposed.

Relationship analysis with other variables.

Pearson correlations were obtained for the relationships among the six-factor scores, and the total score that were obtained for the WHO-QoL-Old scale, the GDS, and the HAS. 

#### 2.3.3. Software

The SPSS 27 program was used for database management and some descriptive analyses, as was the SPSS AMOS program for generating the diagram for the six-correlated factor model. The RStudio program was used to perform the CFA analysis, using the psych, semTools, lavaan, and semPlot packages [24,25,26,27].

## 3. Results

### 3.1. Descriptive Analysis of the Item Scores

Table 2 presents the descriptive statistics (means, standard deviations, skewness, and kurtosis) for each item on the WHO-QoL-Old scale. Additionally, the table includes the Pearson correlations for each item with the scores obtained from the remaining items. All items presented good results, with correlations ranging from 0.299 (Item 9, reversed) to 0.720 (Item 18).

### 3.2. Descriptive Statistics of the Factors and Internal Consistency

Table 3 presents the descriptive statistics and internal consistency results obtained for the factors from the WHO-QoL-Old scale. The six factors exhibit slightly skewed (left-skewed) and slightly platykurtic distributions. The total score obtained for the scale maintains this trend, with a more markedly left-skewed and leptokurtic distribution (skewness: −1.643; kurtosis: 2.730). Favorable internal consistency results were obtained, with an α coefficient ranging from 0.739 to 0.874 and an ω coefficient ranging from 0.748 to 0.882.

### 3.3. Confirmatory Factor Analysis (CFA)

The CFA results, as fit for the models analyzed, are presented in Table 4 and indicate that the best fit is achieved with the six-factor correlated model proposed for the WHO-QoL-Old scale (χ^2^: 418.07, *p* < 0.001; CFI: 0.952; SRMR: 0.081; RMSEA: 0.083). Additionally, the modification indices analysis suggested adding the covariance between the errors of items 1 and 2 (both reversed), which slightly improved the model fit (χ^2^: 397.11, *p* < 0.001; CFI: 0.958; SRMR: 0.079; RMSEA: 0.079).

Figure 1 presents the factor loadings and errors obtained for the fit of the six-factor correlated model. The factor loadings ranged from 0.540 to 0.931, while the correlations between factors obtained by the CFA ranged from 0.275 to 0.861, all statistically significant (*p* < 0.050).

### 3.4. Analysis of Relationships with Other Variables

The Pearson correlations between the six factors, the WHO-QoL-Old scale, and the depression and anxiety scales are presented in Table 5. As can be seen in the table, the GDS results correlated inversely both with all six factors and with the total score for the WHO-QoL-Old scale (with correlations ranging from −0.083 to −0.415), being statistically significant the correlations with the factors 2, 3, and 4. The HAS results also correlated inversely with all six factors and the total score for the WHO-QoL-Old scale (correlations ranging from −0.227 to −0.532), all statistically significant.

## 4. Discussion

The present study has provided evidence of the validity and reliability of the WHO-QoL-Old scale, particularly the Mexican adaptation, which includes pictograms to graphically represent the response options for the items [18,19]. The analysis primarily revealed compliance with the six-factor correlated model proposed, as initially developed for this scale, given that strong correlations were obtained between the items and the rest of the scale, while a satisfactory model fit was obtained via the CFA, as were acceptable internal consistency coefficients (α and ω) for each of the factors.

In contrast with previous studies that tested the internal structure of Spanish versions of the WHO-QoL-Old scale, the present study supports the retention of the six factors originally proposed by the scale developers and of all the items. This contrasts with earlier studies, whose factor solutions suggested eliminating some factors or items [16,17,20]. A similar approach was suggested by the study that originally proposed the scale version that incorporates pictograms in the items [18] and the subsequent Peruvian study [19].

To analyze the relationship with other variables, divergent validity was analyzed with the depression and anxiety scales, obtaining the expected results, namely inverse correlations. This finding concurs with previous studies on the Spanish version of the WHO-QoL-Old scale, which either found an inverse correlation with depression scales or discriminant validity via group comparison tests [14,16,18]. This inverse relationship has also been found in studies conducted in other languages, including the study that developed the original scale [9,11,28,29,30,31]. 

It is important to note that the analysis of divergent validity conducted via the anxiety scale provides new evidence of validity based on relationships with other variables from the Spanish version of the WHO-QoL-Old scale, highlighting that the correlation coefficients obtained were higher than those obtained with the depression scale. Only a few studies conducted on versions of the WHO-QoL-Old scale in other languages have also reported inverse correlations with anxiety scales or mixed depression and anxiety scales [28,32].

Finally, among the strengths of the study, it is worth noting that the recommended data analysis approaches, particularly CFA, were employed in accordance with the recent psychometric research literature. These approaches included the use of an estimator that accounts for the ordinal nature of the items (the WLSMV estimator) [21] and which had not been previously applied on the scale used. However, a weakness of the present study is that it includes a small sample size from a particular group of older adults (residents in nursing homes or members of diurnal care centers).

Also, research opportunities for further studies include evaluating the relationship of the WHO-QoL-Old Scale adapted in Mexico with other scales to test convergent validity like another quality-of-life scale, a life satisfaction scale, a well-being measure, or the participants’ general health status, as well as including other approaches to assess reliability. Other analytical opportunities are the evaluation of measurement invariance as a function of demographic and socio-demographic variables.

## 5. Conclusions

It is concluded that the Spanish version of the WHO-QoL-Old scale, adapted in Mexico and including pictograms for the response options, retains strong psychometric properties and is a viable proposal for a scale that assesses the quality of life in older adults.

## Figures and Tables

**Table 1:** Socio-demographic characteristics of participants (*n* = 111).

	Mean or Frequency
Age in years, M (SD)	77.48 (0.74)
Gender, n (%)	
- Female	81 (72.97)
- Male	30 (27.03)
Type of service, n (%)	
- Nursing Home	70 (63.06)
- Day Care	41 (36.94)
Education, n (%)	
- Illiterate	7 (6.31)
- Primary	28 (25.23)
- Secondary	21 (18.92)
- High School	6 (6.31)
- Technical	37 (33.33)
- Undergraduate	37 (33.33)
- Other	5 (4.50)
Marital status, n (%)	
- Single	23 (20.72)
- Married	43 (38.74)
- Divorced	8 (7.21)
- Widowed	37 (33.33)
Occupation, n (%)	
- Employed	8 (7.21)
- Retired	50 (45.05)
- Homemaker	42 (37.84)
- Religious	9 (8.11)
- Other(s)	2 (1.80)

**Table 2:** Descriptive analysis of the WHO-QoL-Old scale items.

		M	SD	Skewness	Kurtosis	r_total_
	Factor 1. Sensory abilities					
1 *	To what extent do problems with your vision, hearing, taste, smell, and touch affect your daily life?	3.67	0.96	−0.172	−0.620	0.360
2 *	To what extent does the loss of your vision, hearing, taste, smell, and touch affect your ability to participate in activities?	3.97	1.00	−0.494	−0.962	0.469
10 *	To what extent do problems with your vision, hearing, taste, smell, and touch affect your ability to interact with others?	4.10	0.97	−0.807	−0.129	0.422
20	How would you rate the functioning of your vision, hearing, taste, smell, and touch?	3.48	0.903	−0.724	0.216	0.452
	Factor 2. Autonomy					
3	How much freedom do you have to make your own decisions?	3.77	1.084	−0.583	−0.259	0.537
4	To what degree do you feel you have control over your future?	3.13	1.137	0.012	−0.480	0.544
5	To what degree do you feel the people around you respect your freedom?	3.64	1.043	−0.404	−0.423	0.480
11	To what extent can you do the things you would like to do?	3.50	1.035	−0.213	−0.541	0.523
	Factor 3. Past, present, and future activities					
12	To what extent are you satisfied with your opportunities to continue achieving things in life?	3.61	1.089	−0.424	−0.816	0.623
13	To what extent do you feel that you have received the recognition you deserve in life?	3.35	1.109	−0.288	−0.655	0.535
15	To what extent are you satisfied with what you have accomplished in life?	4.01	0.977	−1.210	1.357	0.650
19	To what extent are you content with the things that you can feel enthusiastic about?	3.81	1.005	−0.760	0.008	0.687
	Factor 4. Social participation					
14	To what extent do you feel that you have sufficient activities to engage in each day?	3.24	1.162	−0.064	−0.972	0.607
16	To what extent are you satisfied with the way you use your time?	3.72	1.080	−0.786	−0.089	0.700
17	To what extent are you satisfied with your level of activity?	3.62	1.168	−0.613	−0.759	0.683
18	To what extent are you satisfied with your opportunities to participate in community activities?	3.55	1.150	−0.543	−0.682	0.720
	Factor 5. Death and dying					
6 *	To what extent are you concerned about how you will die?	3.88	1.24	−0.645	−0.857	0.464
7 *	To what extent are you afraid of being unable to control your death?	3.89	1.25	−0.625	−0.996	0.461
8 *	To what extent are you scared of dying?	4.09	1.13	−0.792	−0.774	0.532
9 *	To what extent do you fear experiencing pain before dying?	3.38	1.30	−0.182	−1.201	0.299
	Factor 6. Intimacy					
21	To what extent do you feel a sense of companionship in your life?	3.14	0.949	−0.275	−0.144	0.499
22	To what extent do you experience love in your life?	3.18	1.055	−0.275	−0.423	0.600
23	To what extent do you have opportunities to give love?	3.26	1.150	−0.237	−0.636	0.471
24	To what extent do you have opportunities to receive love?	3.14	1.151	−0.251	−0.732	0.606

Notes: * In these items, the coding was changed, as they are in reverse direction. rtotal: Correlation with the sum of the other items from the scale.

**Table 3:** Descriptive statistics and internal consistency of the factors from the WHO-QoL-Old scale.

	Factor ^a^	M	SD	Skewness	Kurtosis	α Coefficient	ω Coefficient
1	Sensory abilities	15.22	2.91	−0.505	−0.075	0.751	0.764
2	Autonomy	14.04	3.22	−0.156	−0.174	0.739	0.748
3	Past, present, and future activities	14.78	3.28	−0.420	−0.148	0.789	0.779
4	Social participation	14.14	3.79	−0.443	−0.463	0.851	0.858
5	Death and dying	15.24	4.14	−0.626	−0.781	0.861	0.861
6	Intimacy	12.72	3.68	−0.133	−0.637	0.874	0.882
	Total 24-item scale	79.81	25.52	−1.643	2.730	-	-

Notes: ^a^ Scores are calculated considering the reversed coding of items (for Factor 1: items 1, 2, and 10; for Factor 5: all items).

**Table 4:** Pearson correlations between the WHO-QoL-Old, depression, and anxiety scales.

	Factor ^a^	1	2	3	4	5	6	24-Item Scale	Depression Scale	Anxiety Scale
1	Sensory abilities	1								
2	Autonomy	0.427 ***	1							
3	Past, present, and future activities	0.318 ***	0.661 ***	1						
4	Social participation	0.485 ***	0.643 ***	0.761 ***	1					
5	Death and dying	0.283 **	0.199 *	0.420 ***	0.394 ***	1				
6	Intimacy	0.353 ***	0.491 ***	0.545 ***	0.493 ***	0.171	1			
	WHO-QoL-Old scale	0.299 ***	0.322 ***	0.315 ***	0.403 ***	0.250 **	0.405 ***	1		
	Geriatric Depression Scale (GDS)	−0.176	−0.415 **	−0.318 ***	−0.375 ***	−0.083	−0.097	−0.130	1	
	Hamilton Anxiety Scale (HAS)	−0.293 ***	−0.394 ***	−0.501 ***	−0.532 ***	−0.334 ***	−0.261 ***	−0.227 *	0.402 ***	1

Notes: ^a^ Factor scores and WHO-QoL-Old scale considering the reversed coding of items (for Factor 1: items 1, 2, and 10; for Factor 5: all items). * *p* < 0.050; ** *p* < 0.010; *** *p* < 0.001.

**Table 5:** Results of the fit indices obtained from the CFA.

Model	*χ* ^2^	df	CFI	SRMR	RMSEA
1 Factor	1112.85 ***	252	0.773	0.158	0.176
1 Factor Covariances between errors: e6inv-e7inv, e1inv-e2inv, e23-e24	868.57 ***	249	0.837	0.133	0.150
6 Factors	418.07 ***	237	0.952	0.081	0.083
6 Factors Covariances between errors: e1inv-e2inv	397.11 ***	236	0.958	0.079	0.079

*** *p* < 0.001.

## Data Availability

Data can be requested from the corresponding author via email.
